# Automatically transforming pre- to post-composed phenotypes: EQ-lising HPO and MP

**DOI:** 10.1186/2041-1480-4-29

**Published:** 2013-10-16

**Authors:** Anika Oellrich, Christoph Grabmüller, Dietrich Rebholz-Schuhmann

**Affiliations:** 1 European Bioinformatics Institute, Wellcome Trust Genome Campus, Hinxton, Cambridgeshire, CB10 1SD, UK; 2 European Bioinformatics Institute, Wellcome Trust Genome Campus, , Hinxton, Cambridgeshire, CB22 3DQ, UK; 3 Intitut für Computerlinguistik, Universität Zürich, Binzmühlestrasse 14, Zürich, 8050, Switzerland

## Abstract

**Background:**

Large-scale mutagenesis projects are ongoing to improve our understanding about the pathology and subsequently the treatment of diseases. Such projects do not only record the genotype but also report phenotype descriptions of the genetically modified organisms under investigation. Thus far, phenotype data is stored in species-specific databases that lack coherence and interoperability in their phenotype representations. One suggestion to overcome the lack of integration are Entity-Quality (EQ) statements. However, a reliable automated transformation of the phenotype annotations from the databases into EQ statements is still missing.

**Results:**

Here, we report on our ongoing efforts to develop a method (called EQ-liser) for the automated generation of EQ representations from phenotype ontology concept labels. We implemented the suggested method in a prototype and applied it to a subset of Mammalian and Human Phenotype Ontology concepts. In the case of MP, we were able to identify the correct EQ representation in over 52% of structure and process phenotypes. However, applying the EQ-liser prototype to the Human Phenotype Ontology yields a correct EQ representation in only 13.3% of the investigated cases.

**Conclusions:**

With the application of the prototype to two phenotype ontologies, we were able to identify common patterns of mistakes when generating the EQ representation. Correcting these mistakes will pave the way to a species-independent solution to automatically derive EQ representations from phenotype ontology concept labels. Furthermore, we were able to identify inconsistencies in the existing manually defined EQ representations of current phenotype ontologies. Correcting these inconsistencies will improve the quality of the manually defined EQ statements.

## Background

Advances in sequencing technologies have opened up new ways for the systematic exploration of species-specific phenotypic traits linked to selected mutations of a given genome, for example the International Mouse Phenotyping Consortium (IMPC) analyses systematically the mouse genome to this end [1,2]. Phenotype descriptions from such mutagenesis experiments are kept in species-specific Model Organism Databases (MODs) to ensure that the representation of the phenotype data is well-structured in support of further research in comparative phenomics [3]. As the number of available MODs increased [4-6], the same happened to the number of species-specific phenotype ontologies, which nowadays comprise, amongst others, the Mammalian Phenotype Ontology (MP) [7], the Human Phenotype Ontology (HPO) [8] and the Worm Phenotype Ontology (WBPhenotype) [9]. The phenotype ontologies serve as resources for well-chosen and standardised concepts, which support the annotation work. Since the concepts have been prepared prior to the curation work, these ontologies are therefore categorised as pre-composed ontologies. However, these species-dependent phenotype ontologies are very specific to a single species, and thus do not serve well the integration of phenotype data across MODs. In order to facilitate the comparability and exchange of data across all MODs and to support knowledge discovery across all species, other phenotype representations are required.

In principle, there are two ways to achieve interoperability between phenotype ontologies: (1) automatic ontology alignment algorithms, and (2) standardized phenotype representations across all species, i.e. the Entity-Quality (EQ) representation of phenotypes [10]. In the EQ representation each phenotype is represented with an *entity* which is then further described with a *quality*, e.g. *decreased body weight* is composed of the entity *body* which is further specified by the quality *decreased weight*. This approach is called post-composition of phenotype concepts and makes efficient use of existing ontological resources. EQ descriptions have been successfully applied in a number of studies, focusing on cross-species phenotype integration [11-13]. Even though EQ representations are only been used for parts of species-specific phenotype ontologies, selected experiments have already demonstrated beneficial results. However, these studies would certainly profit even more, if more data had been integrated into this framework.

To date, post-composed phenotype representations originate mostly from manual curation work which ensures high quality but is a slow process [14]. Species-specific pre-composed phenotypes are transformed into a post-composed representation by applying the Obol software together with a set of hand-crafted grammar rules required by Obol [15,16]. This automated step is then followed by manual curation step to pick-and-choose the correct EQ statements from the Obol output as well as correcting those EQ statements which are incorrectly formed by Obol. So far, only a subset of the pre-composed phenotype ontology concepts is available as EQ statements (e.g. 4,783 HPO and 6,579 MP concepts). However, a higher coverage of concepts is still required (personal communication with MouseFinder [12] developers) as well as quality improvements to existing EQ statements [14].

Furthermore, any ontology is subject to change reflecting the community effort in capturing the domain knowledge. Concepts evolve, become obsolete or change their representation over time, i.e. the maintenance of the EQ representations consumes effort and updates are a very important requirement. Developing an automated method for the generation of EQ representation from pre-composed phenotype concept would efficiently support the manual curation process, improve quality standards in the maintenance, i.e. reduce curation errors, and enable a higher pace in the ontology development cycle.

In this paper, we present a method (called *EQ-liser*) that transforms pre-composed phenotype ontologies into a post-composed representation using EQ. Our prototype has been applied to MP and HPO concepts to measure its performance and to identify needs for improvement in the process of automatic transformation of pre-composed into post-composed phenotype representations. Our solution not only decomposes pre-composed phenotype labels, but also discovers inconsistencies in manually generated EQ statements and in concept labels from pre-composed phenotype ontologies.

According to our evaluation, our approach generated correct EQ representation for more than 52% of the MP concepts from our test set. We could also identify errors in the existing EQ statements for both HPO and MP, and label inconsistencies within HPO that caused erroneous EQ representations in our approach. Our results, information about the project and the source code are available from our project web page [17].

### Related work

Our gold standard set of EQ statements allowing cross-species phenotype comparisons has been produced by Obol and each EQ statement has been manually curated thereafter [15,16]. Even though the curated EQ statements and the Obol software are accessible, the employed grammar rules required to run Obol are not publicly available. This makes it hard to apply the software to newly created phenotype statements without contacting the authors. Furthermore, no data is available on the number of EQ labels that can correctly be built without the intervention of a curator.

Köhler et al. 2011 [14] emphasised in their study that most EQ statements have been generated manually and pointed out flaws in the existing EQ statements. Therefore, we suggest and provide an open access software solution enabling others to perform quality analyses based on an evaluation file that is generated automatically. We thus support complete transparency of the automated decomposition of phenotype representation and also offer new ways to compare and judge EQ statements from different resources for their overall improvement.

In a recent study, Groza et al. 2012 [18,19] also suggested the decomposition of pre-composed phenotypes, but restricted their study to skeletal phenotypes in human only. The authors use in their approach a corpus of annotated pre-composed phenotype descriptions that contain *entities* and *qualities*. A supervised machine learning algorithm is trained on this corpus and afterwards applied to other pre-composed skeletal phenotypes in order to identify their *entities* and *qualities*. Neither Obol nor EQ-liser apply machine learning in their algorithm. In addition, Groza et al.’s approach does not comply with the logical definitions suggested by Mungall et al. and instead employs a different formalisation to represent post-composed phenotypes [16,18]. We therefore assume that in some cases this leads to different *entities* and *qualities* used to present a certain phenotype. By contrast, our EQ-liser method should comply to the definition of *entities* and *qualities* - as suggested in the original study - with the goal to evaluate the performance of our algorithm with regards to its compliance with the manually assigned EQ statements.

## Results and discussion

Transforming a pre-composed into a post-composed phenotype representation requires an analysis of the concept labels to identify the affected *entity* and corresponding *qualities* relevant to a particular phenotype. The *entities* as well as the *qualities* have to be matched to ontological concepts that are provided from other OBO Foundry ontologies. As use case scenario, we have tested the *EQ-liser* method on MP and HPO concept labels. Note that all decomposition attempts are only executed on structure and process phenotypes.

### *EQ-lising* the mammalian phenotype ontology

3,549 concept labels (out of 3,761) could be transformed when processing the concept labels of MP’s structure and process phenotypes. Comparing these to our gold standard EQ statements shows that 23.7% had been assigned a correct post-compositional representation by *EQ-liser*. Exploiting synonyms in addition, we could improve our results by 6.7%. If we allow *EQ-liser* to assign more annotations than a manual curator would do, i.e. we take a larger number of automatically generated EQ representation into consideration, we achieve to identify *entities* together with their *qualities* that are correct for 52.2% of MP concepts. We believe that the relaxing performance assessment is reasonable, since all generated EQ statements will be evaluated by a curator and additionally assigned *entities* or qualities (apart from the *entity* and the *quality* required to represent the phenotype) could be removed without much effort, if required. Automatically deriving an EQ representation for more than half of MP’s structure and process phenotypes, is a very promising achievement for our generalised decomposition method. Erroneous and thus useless representations of post-composed phenotype concepts have only been generated for 5.6% of the concepts. These numbers indicate that the pre-composed concept labels of MP are already well formed and that the automatic transformation – with a grain of salt – does generate post-composed representations that correctly reflect the semantics of the pre-composed representation.

#### Mismatches in *EQ-lising* MP

We then selected 50 MP concepts where the automatically derived EQ representation and the manually assigned EQ statements did not match. We manually compared both EQ representations and identified the reasons for the mismatch. This lead to the discovery of the following shared patterns with regards to the three components of the EQ representations (structure, process, and quality).

A number of mismatches were caused by assigning wrong PATO annotations due to particular extension or replacement patterns in the manually designed EQ statement which cannot yet be picked up with the automated procedure. For example, the automatically generated EQ statement *quality* of *increased mitochondrial proliferation* (MP:0006038) corresponds to *increased rate* (PATO:0000912) from the manually assigned EQ statements. However, the automated method chooses *increased* (PATO:0000470) as *quality* for this particular MP concept. In the same vein, all concept names containing the phrase *increased activity* have been annotated with *increased rate* (PATO:0000912) in the manually assigned EQ statements which cannot be reproduced with the automatic method. Furthermore, every phenotype concept with the phrase *increased... number* in their label, possesses the quality *has extra parts of type* (PATO:0002001) in the manually assigned EQ statement. The same examples can be found if the term *increased* in the concept label is replaced with *decreased*. All our examples could be resolved by introducing conditional replacement rules for PATO concepts, which in return would lead to a reduction of the contradictory cases and to an increase in the number of correctly identified EQ representations.

Further mismatches resulted from missed or faulty identification of the structure *entity* in the phenotype representation, for example when the affected anatomical structure is named differently in Mouse Anatomy Ontology (MA) [20] and MP. Often this is due to singular/plural divergence, e.g. the MA concept label *lumbar vertebra* (MA:0000312) cannot be automatically attributed to the MP concept *increased lumbar vertebrae number* (MP:0004650) since *vertebra* and *vertebrae* differ morphologically. Moreover, mismatches occurred when short forms for anatomical structures were used, e.g. MP simply uses *coat* while MA mentions *coat hair*. These mismatches could be addressed by augmenting the dictionary in the LingPipe [21,22] MA annotation server or by applying a stemming to both concept labels and synonyms, and the underlying annotation dictionary.

The third type of mismatches occurs in the process *entity* of the EQ representations. Mismatches partially resulted from a lack of synonyms in the current GO annotation server. For example, concept names including the process entity *salivation* were not recognised as the process *saliva secretion* contained in GO. In other cases, different word forms for a concept caused problems, e.g. *smooth muscle contractility* and *smooth muscle contraction*. Again singular and plural variability caused mismatches in the process constituent, e.g. MP makes use of *cilia* while GO applies *cilium* representing the plural and singular of *cilium*, respectively. The synonym mismatches and singular/plural-conflicts can be resolved by larger dictionary resources and the integration of stemming prior to the entity recognition step.

In two out of all 50 evaluated concepts, we could identify an erroneously, manually assigned EQ statement in our gold standard (corresponds to 4% of the investigated cases), which have been reported to the curation team for correction. The errors mainly resulted from older construction patterns in combination with concepts that have been recently added to the constituent ontologies.

### *EQ-lising* the human phenotype ontology

Then we determined the transformation performance of our solution on another pre-composed phenotype ontology, i.e. we applied *EQ-liser* to the HPO concept labels. HPO has been selected, since it serves as ontology for another mammal species, and we expect that both ontologies, i.e. HPO and MP, share similar phenotype concepts. Our analysis was again limited to structural and process phenotypes only. We used concepts from the Foundational Model of Anatomy (FMA) ontology [23], the Gene Ontology (GO) [24] and PATO to build post-composed phenotype representations.

We analysed 3,268 pre-composed concepts, of which 2,731 have obtained an automatically assigned EQ representation. Only 231 (8.5%) generated EQ representations showed an exact match to the manually assigned EQ statements. If we include synonyms, we can increase the matching cases to a total of 249 (9.5%). If we then relax the matching criterion, i.e. allow additionally assigned *entities* or *qualities* in EQ representations, we obtain correct annotations in 13.3% of the cases. In 25.8% of all cases, none of the manually assigned *entities* or *qualities* could be reproduced by *EQ-liser*. Our results demonstrate that the decomposition of mouse phenotype concepts can be achieved at a higher rate using lexical features and synonyms, in contrast to the human counterparts.

#### Mismatches in *EQ-lising* HPO

One reason for the mismatches with regards to the quality in the phenotype representation is again the term variability in the quality description. For example, HPO concepts containing either *abnormality* or *abnormalities* do not receive the quality *abnormal* (PATO:0000460) automatically due to the morphological variability of the terms. Furthermore, all concepts with reference to *abnormality* or *abnormalities* possess the manually assigned quality *quality* (PATO:0000001) which cannot be derived automatically from the pre-composed concept. Moreover, some terms contained in HPO concept labels are further specified in the manually assigned EQ statement. For example, the term *irregular)* in *Irregular epiphysis of the middle phalanx of the 4th finger* (HP:0009219) is translated into *irregular density* (PATO:0002141) in the manual assignment. Such mismatches can be corrected by adding special transformation rules in the concept decomposition step, which would be specific for HPO.

Mismatches in the representation of structure *entities* in HPO phenotypes were partially due to diverging naming conventions in HPO and FMA, e.g. while FMA calls fingers with a name (*index finger* or *ring finger*), HPO assigns numbers to fingers, such as *2nd finger* or *fourth finger*. However, HPO does not apply the numbering consistently across all concepts concerned with digits, e.g the expression *thumb* is used where the *first finger* is concerned. Furthermore, HPO is not well standardised with regards to singular and plural usages of nouns, e.g. (*phalanges* versus *phalanx*). Mismatches also result from the introduction of contractions used in HPO concept labels while FMA uses full descriptions, e.g. *premolar* instead of *premolar tooth* or *metatarsal* instead of *metatarsal bone*. Most of these mismatches can be resolved by augmenting the dictionary of the LingPipe FMA annotation server with additional terms.

Analoguous to mismatches in MP (see section “*Mismatches in EQ-lising MP*”), mismatches in process *entities* were partially due to not supporting synonyms in the current implementation of the GO server. For example, *Abnormality of valine metabolism* (HP:0010914) does not obtain the GO annotation *valine metabolic process* (GO:0006573). Such mismatches can be corrected in future versions of the *EQ-liser* method by including synonyms in the current version of the GO annotation server.

The last type of mismatches occurred rarely and only when decomposing HPO labels: identical concepts co-exist in different ontologies, i.e. not all ontologies are orthogonal although OBO Foundry strives for this goal. For example, both FMA and GO contain the concept *Chromosome* (GO:0005694, FMA:67093) and the developer of the manually assigned EQ statements is free to choose either one. This consequently leads to inconsistencies in automated decomposition methods. Another example for the duplication of a concepts is *Anosmia* (HP:0000458, PATO:0000817). These concepts should be removed during the process of quality assessment through the OBO Foundry, whereas the decomposition method may well ignore this aspect. We found this mismatch in three concepts (6% of the investigated cases). These inconsistencies were reported to, confirmed and corrected by the HPO EQ statement developers and are now available.

### Towards a generalised phenotype decomposition

Even though the automated decomposition of HPO concepts lags behind the automated generation of EQ representations for MP concepts with the *EQ-liser* method, the error analyses for either ontology is similar and improving the approach would resolve the mismatches for both ontologies alike. Achieving 52% performance for the structural and process phenotypes in MP is a good start for the automated transformation of pre-composed labels from a phenotype ontology into a post-composed representation. However, under the consideration that EQ statements for MP and HPO have been developed in a collaborative way and in close range, our method has to be further validated on other pre-composed phenotype ontologies. We expect that the performance of our proposed method will increase once the main mismatches have been addressed and further validation has been performed. We aim to provide a precise automated decomposition of phenotype labels for all species under the condition that relevant ontologies for *entities* and *qualities* are available.

## Conclusions

*EQ-liser* generates EQ representations for structural and process phenotypes from MP and yields correct results in 30% of the cases under strict measures, and 52% under relaxed measures. In the latter case we assume that we produce a larger set of annotations under the consideration that a curator will manually assert and approve the EQ representation before they are used community wide, and will remove incorrect assignments. The decomposition of HPO labels can only be achieved at a lower rate until solutions for a number of identified problems have been implemented. Addressing these problems should also lead the way to a generalised approach for the automated generation of EQ representations from pre-composed phenotype labels. Altogether we will achieve interoperability between species-specific databases containing phenotypic descriptions of model organisms.

Apart from decomposing pre-composed phenotype concept labels, our method is also capable of identifying inconsistencies in the composition of the pre-composed labels. While MA and MP follow a rigorous naming scheme and hence support integration based on concept labels, FMA and HPO differ in their naming conventions creating obstacles for all data integration efforts. Furthermore, HPO shows internal inconsistencies in its naming conventions, which have to be removed for better interoperability.

Furthermore, we could identify flaws in the manually assigned EQ statements by systematically comparing them against the automatically generated representations. We thus improved the quality of the existing EQ statements and consequently also the performance of all methods applying these, e.g. PhenomeNET [13] or MouseFinder [12].

In the future, we aim to cover all phenotypes contained in existing pre-composed phenotype ontologies. Our solution will be made available to the research community as a web interface and a command line tool.

## Methods

Transforming pre-composed phenotype representations into post-composed ones requires the identification of *entities* and *qualities* in concept labels. To illustrate the post-composition of the MP concept *abnormal otolithic membrane* (MP:0002895), the manually assigned EQ statement is provided here:


### Input data

In the existing, manually derived EQ statements, an *entity* is represented with a number of OBO Foundry ontologies [25] and a *quality* is always represented using the Phenotypic quality And Trait Ontology (PATO) [10,26]. *Entity* filling ontologies also differ with the species. Supporting all ontologies would be beyond the scope of this study. We therefore limited our approach to two species-specific ontologies, HPO and MP. More specifically, we only included phenotype concepts represented in the manually assigned EQ statements with: the Mouse Anatomy Ontology (MA) [20], the Gene Ontology (GO) [24], the Foundational Model of Anatomy Ontology (FMA) [23] and PATO. We consider this to be corresponding to structural and process phenotypes. We downloaded a version of the two phenotype ontologies as.tbl files [27] and their corresponding EQ statements on the 03.05.2012, with 9,795 HPO concepts and 9,127 MP concepts. 4,783 HPO and 6,579 MP concepts possess a manual assigned EQ statement. We note here that our method so far only supports structure and process phenotypes and therefore reduced the number of concepts we apply our method to based on the manually assigned EQ statements. The reduced data set comprises 3,761 MP and 3,268 HPO concepts with their corresponding manually assigned EQ statement.

### Deriving PATO cross products

A subset of the PATO concepts constitute a composition of other PATO concepts. For instance, the concept *decreased depth* (PATO:0001472) could be represented using the PATO concept *decreased* (PATO:0001997) and *depth* (PATO:0001595). To achieve a term-wise composition of PATO concepts, we downloaded the PATO.tbl file and applied the filtering and stemming algorithm as described in section “*Overview EQ-liser prototype*”. The composition of one particular PATO concept corresponds to all PATO concepts whose terms form a subset of the stemmed words contained in the concept name.

After filtering special characters and removing stop words from the concept names and synonyms, the remaining textual content was stemmed using a Porter stemmer [28] provided by Snowball [29]. The stemmer was applied to all concept names and synonyms. Stemmed concept labels and synonyms were then pairwise compared and each concept entirely contained in another (either label or synonym) was recorded. Applying this process we retrieved 1,453 PATO concepts (out of 2,290) with a corresponding cross product.

### Overview *EQ-liser* prototype

**Figure 1:**
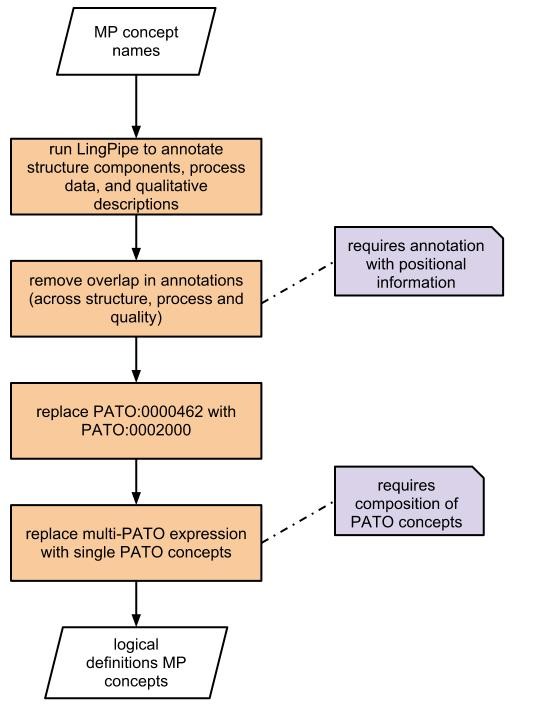
***EQ-liser*’s workflow.** Shows the individual steps executed with *EQ-liser* to decompose a phenotype ontology based on concept names.

Figure 1 shows the processing steps to derive the EQ representation from a MP or HPO phenotype concept. Each of the steps is explained in more detail in the following paragraphs.

The first step (see Figure 1) in processing the ontology’s downloaded.tbl file was the filtering for special characters. Therefore, the concept labels contained in the downloaded.tbl files^a^ of the ontologies were analysed for their orthographic correctness [30], i.e. special characters, such as e.g. “%” or “-”, were excluded. Such special characters – often special punctuation – potentially cause problems when matching differently punctuated concept labels from several ontologies. Stop words, such as “in” or “the” are part of the common English language, considered not to carry any discriminatory information and consequently can be removed before analysis to reduce noise and potential errors resulting from their inclusion.

After character filtering and stop word removal from all the concept labels and their synonyms, we used LingPipe [21] to recognise entities and qualities from MP and HPO concepts. The dictionaries for LingPipe were compiled by using the labels and synonyms provided by the ontology files for FMA, MA and PATO. For GO, we used an alternative approach described in [31] but also implemented as LingPipe annotation server. A single tagging server has been established for each ontology. All servers work parallel and may assign overlapping annotations which could potentially result in too many annotations assigned by the automated method. E.g. in the case of *enlarged dorsal root ganglion* (MP:0008490), an MA annotation for *dorsal root ganglion* (MA:0000232) and a PATO annotation for *dorsal* (PATO:0001233) is assigned. To avoid this behaviour, we ran a filter process after assigning LingPipe annotations and removed all annotations that are entirely included in others. Filtering GO annotations is not yet possible due to the current implementation of this server but will be supported in later versions.

In the last step we automatically replaced LingPipe’s PATO annotations and combined them into cross products representation where possible (see section “*Deriving PATO cross products*” for further details). We note here that not all PATO annotations are necessarily combined, only those for which we identified a cross product before. Consequently, in the before mentioned example of *decreased palatal depth*, the two LingPipe annotations would be replaced now with one single annotation *decreased depth*. In addition, *absent* (PATO:0000462) is replaced in all automated EQ statements with *lacks all parts of type* (PATO:0002000) which is commonly used in the manual assigned EQ descriptions.

### Evaluation

To evaluate our results, we introduced a two-step evaluation process. We first evaluated the obtained EQ representation to the available, manually assigned EQ statements of structural and process phenotypes. In a second step, we investigated a subset of 50 EQ representations of each ontology where automated method and manual curator do not assign any shared concepts. Common patterns were identified causing disagreements in the automatically assigned EQ representation and are discussed in sections “*Mismatches in EQ-lising MP*” and “*Mismatches in EQ-lising HPO*”, for MP and HPO respectively.

## Endnote

^a^ provides a tabular view an ontology’s data; generated from.obo files.

## Abbreviations

EQ: Entity-quality; FMA: Foundation model of anatomy; GO: Gene ontology; HPO: Human phenotype ontology; IMPC: International mouse phenotype consortium; MA: Adult mouse anatomy ontology; MOD: Model organism databases; MP: Mammalian phenotype pntology; OBO: Open biological and biomedical ontologies; PATO: Phenotype and trait quality ontology.

## Competing interests

The authors declare that there are no scientific as well as financial competing interests.

## Authors’ contributions

AOE outlined the project and designed the experimental set-up. AOE and CHG implemented the required scripts and server set-up. DRS supervised the study. All contributed to the manuscript. All authors read and approved the final manuscript.
